# Vasoactivity of Rac GTPase, Cytohesin and Kinase Inhibitors in Renal Interlobar and Coronary Arteries Reveals Shared and Distinct Patterns of Inhibitory Effects in Vascular and Prostate Smooth Muscle Contraction

**DOI:** 10.1002/prp2.70190

**Published:** 2025-11-18

**Authors:** Guangyang Liu, Sheng Hu, Alexander Tamalunas, Oluwafemi Kale, Yajie Xu, Christian G. Stief, Martin Hennenberg

**Affiliations:** ^1^ Department of Urology, LMU University Hospital LMU Munich Munich Germany

**Keywords:** benign prostatic hyperplasia, lower urinary tract symptoms, smooth muscle contraction, vacocontraction, voiding symptoms

## Abstract

Inhibition of vasocontraction accounts for side effects in treating voiding symptoms in benign prostatic hyperplasia (BPH). We examined the vasoactivity of compounds previously showing inhibition of prostate smooth muscle contraction. Contractions of porcine renal interlobar and coronary arteries were induced by agonists or electric field stimulation (EFS). Examined compounds included inhibitors for Rac GTPases (EHT1864, NSC23766), cytohesin GEFs (SecinH3), LIMK (SR7826, LIMKi3), βARKs (CMPD101), PAK (FRAX486), and ILK (Cpd22). Agonist‐ and EFS‐induced contractions in renal and coronary arteries were completely inhibited by 100 μM EHT1864, and nearly completely at 10 μM. In renal arteries, 100 μM NSC23766 right‐shifted concentration response curves (increased EC_50_) for α_1_‐adrenergic agonists, halved U46619‐induced and fully inhibited EFS‐induced contractions. Right shifts (increased EC_50_) for phenylephrine still occurred at 10 and 1 μM. In coronary arteries, 100 μM NSC23766 produced right shifts (increased EC_50_) for cholinergic agonists. SecinH3 (30 μM) reduced cholinergic contractions in coronary but not renal arteries. In renal arteries, SR7826, but not LIMKi3 (both 1 μM), partly inhibited (< 50%) agonist‐ and EFS‐induced contractions. CMPD101 (50 μM) inhibited (≥ 50%) α_1_‐adrenergic and U46619‐induced contractions, but no endothelin‐1‐ or EFS‐induced contractions. Neither FRAX486 (30 μM), nor Cpd22 (3 μM) affected contractions. Vasorelaxation by EHT1864 and NSC23766 may exclude application in BPH but may allow simultaneous treatment of cardiovascular disease and BPH. NSC23766 shows previously unrecognized α_1_‐adrenoceptor antagonism. Findings with SecinH3 suggest an organ‐selective involvement of cytohesin‐2/Arf6 signaling in smooth muscle contractions. SR7826 may cause cardiovascular effects, while side‐effect risks limit kinase inhibitors in non‐malignant disease.

## 1 Introduction

Smooth muscle contraction is the target for medical treatment of voiding symptoms in benign prostatic hyperplasia (BPH) [1, 2], and of antihypertensive drugs. Voiding symptoms in BPH may be caused by increased prostate smooth muscle tone [1, 2]. Available drugs include α_1_‐adrenoceptor antagonists (“α_1_‐blockers”) as the first‐line option, and the phosphodiesterase‐5 inhibitor tadalafil, which are both believed to improve symptoms by prostate smooth muscle relaxation [3]. However, average improvements are limited to 50% and one third of treated patients are non‐responders [1], calling for newer drugs. The tolerability and patients' adherence to α_1_‐blockers are limited by cardiovascular side effects, whereas the application of tadalafil is restricted by cardiovascular contraindications [3, 4]. Based on the physiological and pharmacological similarities of vascular and prostate smooth muscle contractions, vasoactive effects would play a role in the development of new drug candidates.

Voiding symptoms and hypertension are common comorbidities in elderly patients. Combinations of α_1_‐blockers with antihypertensives are the most prescribed drug cluster in men aged ≥ 70 years [5], although α_1_‐blocker‐induced hypotension is most prevalent with cardiovascular comorbidities and in co‐medication with cardiovascular drugs [4, 5]. Accordingly, multidrug regimens inherently carry the risk of additive side effects [6, 7]. On the other hand, new single compounds with simultaneous effects on voiding symptoms and vasocontraction may theoretically help to reduce polypharmacy, again highlighting the need to understand the shared and distinct impact on vascular and prostate smooth muscle contractions.

Drug‐resistant voiding symptoms have been attributed to non‐adrenergic prostate smooth muscle contractions, which are insensitive to α_1_‐blockers [1, 8, 9]. Recently, several compounds have been identified that inhibit endothelin‐ and thromboxane‐induced as well as adrenergic contractions in human prostate tissue. Compounds with this effect profile included small molecule inhibitors for Rac GTPases [10, 11], cytohesin family guanosine exchange factors (GEF) [12], LIM kinases (LIMK) [13], beta‐adrenergic receptor kinases (βARKs, syn. G protein‐coupled receptor kinases 2 and 3, GRK2, ‐3) [14], p21‐activated kinases (PAK) [11, 15], and integrin‐linked kinase (ILK) [16]. For a first assessment of their potential vasoactivity, we here examined the effects of EHT1864 and NSC23766 (RacGTPase inhibitors), secin H3 (cytohesin inhibitor), SR7826 and LIMKi3 (LIMK inhibitors), CMPD101 (GRK2/3 inhibitor), FRAX486 (PAK inhibitor), and Cpd22 (ILK inhibitor) on contractions of porcine renal interlobar arteries and partly in coronary arteries.

## 2 Methods

### 2.1 Porcine Renal Interlobar and Coronary Arteries

Porcine kidneys and hearts were obtained from a local slaughterhouse, where pigs were sacrificed for meat production at night. Organs were collected by a butcher the same night, stored at 4°C and transported from the butcher's shop (Metzgerei Brehm, Planegg, Germany) to the nearby laboratory in the early morning. Segments of renal interlobar or coronary arteries were prepared from organs immediately after arrival in the laboratory. Connective and adipose tissue surrounding the vessels was removed, and segments were cut into rings (3–4 mm in length), which were stored in Custodiol solution (Köhler, Bensheim, Germany) at 4°C until use in experiments. Experiments were started within 3 h following vessel preparation.

### 2.2 Organ Bath Experiments

Vessel rings with a length of 3–4 mm were mounted in myograph systems, with four chambers each (model 720 M; Danish Myo Technology, Aarhus, Denmark). Each chamber contained 10 mL Krebs–Henseleit solution (37°C, pH 7.4), which was continuously aerated with carbogen (95% O_2_ and 5% CO_2_) during the experiment. The rings were placed to surround the two opposing needles in the interior of the chambers, and a passive pretension of 10 mN was applied to segments of renal arteries, and 20 mN to segments of coronary arteries. Since pretension typically declines shortly after mounting, it was readjusted during the following 45 min to reach a stable baseline tension. For later reference of agonist‐ and electric field stimulation (EFS)‐induced contractions to high molar potassium‐induced contractions, rings were subsequently contracted by addition of a 2 M KCl solution to the chambers, resulting in a final potassium concentration of 80 mM. As soon as a plateau or maximum contraction was reached, the solution in the chambers was washed three times with fresh Krebs–Henseleit solution within 30 min. After a new, stable baseline was obtained, inhibitors or solvent (control groups) were added. Cumulative concentration response curves for agonists or frequency response curves for EFS were constructed after a further 30 min.

The initial study plan included experiments in renal arteries using the highest concentrations of test compounds (i.e., 100 μM EHT1864 and NSC23766, 30 μM SecinH3 and FRAX486, 1 μM SR7826 and LIMKi3, 50 μM CMPD101, and 3 μM Cpd22). After completion of these initial experiments and following data analyses, additional experiments were carried out addressing the effects of lower concentrations of EHT1864 and NSC23766 (10 and 1 μM) on phenylephrine‐ and EFS‐induced contractions in renal arteries, and addressing the effects of EHT1864 (100, 10, and 1 μM), NSC23766 (100, 10, and 1 μM) and SecinH3 (30 and 10 μM) in coronary arteries. Unlike in the initial experiments, experiments with tissues showing only weak KCl‐induced contractions (< 0.5 mN) were not excluded from subsequent experiments in coronary arteries or with descending concentrations, resulting in higher variations of agonist‐ and EFS‐induced contractions.

The rings for control and inhibitor groups within an independent experiment were obtained from the arteries of the same animal. Each experiment was repeated five times per series, so that five experiments with paired rings of five animals were performed per series. Only one concentration response or frequency response curve was recorded with each sample. Each experiment intended double determination in both groups, that is, included two rings for the control group and two rings for the inhibitor group in the beginning, with all four rings obtained from the same artery. In the initial experiments with the highest examined concentrations of test compounds in renal arteries, duplicate determinations were consistently maintained, except for three single experiments as follows. One ring per experiment had to be excluded as it detached from the needles in the EHT1864 group (100 μM) with U46619, or because the electrode accidentally touched the tissue in the NSC23766 group (100 μM) with EFS, or because the tissue was fully unresponsive to KCl in the control group of an experiment addressing the effects of LIMKi3 on phenylephrine. Consequently, values in these groups were based on single determinations. In additional experiments addressing the effects of lower concentrations in renal arteries or effects on coronary arteries, the failure rates were higher (32 of 100 experiments were based on single determination in one of the both groups), but each experiment included at least data from one channel (i.e., one group). Allocations of channels to control and inhibitor groups were changed between experiments. Agonist‐ and EFS‐induced contractions are expressed as a percentage of 80 mM KCl‐induced contractions, as this may correct any individual variations resulting from tissue size, technical procedures, or from individual heterogeneities in animals.

### 2.3 Curve Fitting

*E*_max_ values, EC_50_ values for agonists, and frequencies (*f*) inducing 50% of the maximum EFS‐induced contraction (EF_50_) were calculated separately for each single experiment by curve fitting as previously described [17], using GraphPad Prism 6 (GraphPad Software Inc., San Diego, CA, USA). The software sends error messages, if curve fitting is not possible or if results from curve fitting are suspected as “ambiguous” (i.e., as non‐plausible). In addition, values from curve fitting were checked manually for plausibility, as recommended in the “GraphPad Curve Fitting Guide” (i.e., the manufacturer's instructions) (GraphPad Software Inc., San Diego, CA, USA). Several values marked as “ambiguous” (mostly, though not exclusively in U46619 experiments) appeared plausible after manual review, despite being labeled as “ambiguous.” As further recommended in the curve fitting guide, downhill parts of concentration response curves were excluded from curve fitting (but not in diagrams showing whole concentration response curves) in the initial experiments (addressing the highest concentrations of test compounds in renal arteries), if curve fitting was otherwise not possible, or if the results were otherwise not plausible. Despite apparent nearly complete inhibitions by EHT1864, curve fitting was still possible in most of these experiments, partly, however, because the software detected declines from the baseline pretension during the experiment. Nevertheless, the resulting *E*_max_ values have been included here as they virtually reflect the extent of inhibition seen in concentration and frequency response curves, while the resulting EC_50_ values are of low or lacking validity but are reported for completeness only. Exceptions in initial experiments with the highest applied concentrations in renal arteries were one experiment with EHT1864 and phenylephrine, one with EHT1864 and EFS, and one with EHT1864 and noradrenaline, where calculation was not possible due to complete inhibition. In these experiments, the highest contractions from the curves (all close to zero) were taken as *E*_max_ values and are marked in gray in corresponding scatter plots, and the highest applied agonist concentration (log M −3) and the highest applied frequency (32 Hz) were used as approximations for the EC_50_ and EF_50_. Similarly, curve fitting was impossible in the inhibitor group of one experiment addressing NSC23766 effects and one addressing SR7826 effects on endothelin‐1‐induced contractions, so that again the highest contraction in the curve was taken as the *E*_max_, whereas any reasonable estimation of an approximated EC_50_ was impossible in the NSC23766 experiment, but the highest endothelin‐1 concentration (log M −5.5) was applied as an approximation for the EC_50_ in the SR7826 experiment. In additional experiments addressing lower concentrations of EHT1864 and NSC23766 in renal arteries, and of EHT1864, NSC23766, and SecinH3 in coronary arteries, downhill parts of concentration response curves were generally excluded in curve fitting. Analog to initial experiments, implausible values or *E*_max_ values for curves that could not be calculated by curve fitting were replaced by maximum tensions seen in curves, and EC_50_ values were estimated from curves. Values from these procedures have been marked gray in scatter plots.

### 2.4 Calculation of Apparent pA_2_ Values

To estimate the affinity of NSC23766 for α_1_‐adrenoceptors in our experiments, “apparent” pA_2_ values were calculated as the sum of the negative decadic logarithm of the NSC23766 concentration, and the right shift in concentration response curves for α_1_‐adrenoceptors, expressed as negative decadic logarithm: apparent pA_2_ = p[NSC23766] + (pEC_50_ α_1_‐agonist controls—pEC_50_ α_1_‐agonist with NSC23766). Values were calculated separately for each single experiment. The structure of our data differs from that required to calculate exact pA_2_ values. The calculation of true pA_2_ values requires determinations with several ligand concentrations and a single, shared control group within the same experiment. However, our experimental design was based on two groups per experiment, involving only one NSC23766 group and a control group, which does not allow calculation of real pA_2_ values. Accordingly, the interpretation of apparent pA_2_ values may be subject to limitations, but can be used as an approximation to a pA_2_ value or an estimate of affinity [18, 19]. Calculations of pA_2_ values were limited to initial experiments with the highest tested concentrations in renal arteries, while additional experiments addressing lower concentrations and effects in coronary arteries were excluded from the calculation of apparent pA_2_ values, for limitations declared above (higher rate of failure in single channels, and higher variations of agonist‐induced contractions).

### 2.5 Drugs and Nomenclature

Phenylephrine ((R)‐3‐[−1‐hydroxy‐2‐(methylamino)ethyl]phenol) and methoxamine (α‐(1‐aminoethyl)‐2,5‐dimethoxybenzyl alcohol) are α_1_‐selective adrenoceptor agonists [20]. U46619 is commonly used as a TP receptor (thromboxane A_2_ receptor) agonist, as thromboxane A_2_ is highly unstable [20]. Carbachol (2‐carbamoyloxyethyl‐trimethylazanium) and methacholine (2‐acetyloxypropyl‐trimethylazanium) are muscarinic receptor agonists [20]. The examined test compounds included inhibitors related to signaling by monomeric GTPases (Table 1) and kinase inhibitors (Table 2), which previously inhibited contractions of human prostate tissues. All inhibitors were used here at the same concentrations as previously with prostate tissues (Tables 1 and 2). EHT1864 (2‐(morpholin‐4‐ylmethyl)‐5‐[5‐[7‐(trifluoromethyl)quinolin‐4‐yl]sulfanylpentoxy]pyran‐4‐one) is an inhibitor of Rac family GTPases, blocking/inhibiting Rac1, Rac1b, Rac2, and Rac3 with *K*_d_ values of 40, 50, 60, and 250 nM, respectively [21, 22]. NSC23766 (N′‐[2‐(5‐diethylaminopentan‐2‐ylamino)‐6‐methylpyrimidin‐4‐yl]‐2‐methylquinoline‐4,6‐diamine) is a selective inhibitor of Rac1‐GEF interaction, by preventing Rac1 activation by the Rac‐specific GEFs TrioN and Tiam1 with an IC_50_ about 50 μM, without affecting Cdc42 or RhoA activation [23, 24, 25]. However, off‐targets include antagonism of muscarinic receptors [26, 27]. SecinH3 (N‐[4‐[5‐(1,3‐Benzodioxol‐5‐yl)‐3‐methoxy‐1H‐1,2,4‐triazol‐1‐yl]phenyl]‐2‐(phenylthio)acetamide) is a cytohesin family inhibitor, with IC_50_ values of 2.4, 5.4, 5.4, 5.6, 5.6, 65, and > 100 μM for human cytohesin‐2, human cytohesin‐1, mouse cytohesin‐3, human cytohesin‐3, Drosophila steppke, yGea2‐S7, and hEFA6‐S7 [28, 29]. SR7826 (1‐(2‐hydroxyethyl)‐3‐[4‐(5‐methyl‐7H‐pyrrolo[2,3‐d]pyrimidin‐4‐yl)phenyl]‐1‐phenylurea) inhibits LIMK1 with an IC_50_ of 43 nM, and Rho kinase 1 (ROCK1) and Rho kinase 2 (ROCK2) with IC_50_ values of 5536 and 6565 nM [30]. LIMKi3 (N‐[5‐[2‐(2,6‐dichlorophenyl)‐5‐(difluoromethyl)pyrazol‐3‐yl]‐1,3‐thiazol‐2‐yl]‐2‐methylpropanamide) inhibits LIMK1 with an IC_50_ of 7 nM and LIMK2 with an IC_50_ of 8 nM [31]. CMPD101 (3‐[(4‐methyl‐5‐pyridin‐4‐yl‐1,2,4‐triazol‐3‐yl)methylamino]‐N‐[[2‐(trifluoromethyl)phenyl]methyl]benzamide) inhibits GRK2 (beta adrenergic receptor kinase 1) with reported IC_50_ values of 35 nM, 54 nM or 290 nM, and GRK3 (beta adrenergic receptor kinase 2) with an IC_50_ of 32 nM [32, 33]. Screening of 58 kinases representative of the entire kinome (though excluding lipid kinases) identified five off‐target kinases inhibited by CMPD101 by > 50% using 1 μM, including protein kinase N2 (PKN2, syn. PRK2), serum/glucocorticoid regulated kinase 1 (SGK1), ribosomal protein S6 kinase A1 (RSK1), ribosomal protein S6 kinase A5 (MSK1), and ribosomal protein S6 kinase B1 (p70S6K, syn. S6K1) [34]. FRAX486 (6‐(2,4‐dichlorophenyl)‐8‐ethyl‐2‐(3‐fluoro‐4‐piperazin‐1‐ylanilino)pyrido[2,3‐d]pyrimidin‐7‐one) is an inhibitor for group 1 PAKs, with IC_50_ values of 8.25 nM for PAK1, 39.5 nM for PAK2, 55.3 nM for PAK3, and 779 nM for PAK4 [35]. Cpd22 (N‐methyl‐3‐[1‐(4‐piperazin‐1‐ylphenyl)‐5‐[4‐[4‐(trifluoromethyl)phenyl]phenyl]pyrazol‐3‐yl]propanamide, “compound 22”) inhibits ILK with an IC_50_ of 0.6 μM [36]. Using 5 μM, 17 of 20 further screened kinases were inhibited by not more than 10%, while two were inhibited to 27% (Abl and CDK1) and one by more than 50% (p70S6K) [36]. Using a concentration of 5 μM, Cpd22 inhibited the activities of 17 of 20 screened kinases by not more than 10%, while only three were inhibited to 27% (Abl and CDK1) or by more than 50% (p70S6K) [36]. Noradrenaline, phenylephrine, and methoxamine were obtained from Sigma‐Aldrich (Munich, Germany). EHT1864, NSC23766, SR7826, LIMKi3, CMPD101, FRAX486, and SecinH3 were obtained from Tocris (Bristol, UK). Cpd22 was obtained from Merck (Darmstadt, Germany). Aqueous stock solutions (10 mM) of noradrenaline, phenylephrine, and methoxamine were freshly prepared before each experiment. Stock solutions (10 mM) of EHT1864 and NSC23766 were prepared with deionized water, of SR7826, LIMKi3, Cpd22, FRAX486, and SecinH3 with dimethylsulfoxide (DMSO), and of CMPD101 with ethanol, and stored as aliquots at −20°C until use.

**TABLE 1:** Inhibitors for GTPase signaling used in this study.

	IC_50_ biochemical assays		Prostate tissues		Renal interlobar arteries		Coronary arteries	
	Target	Off‐target	Applied concentration (μM)	Outcome	Applied concentration (μM)	Outcome	Applied concentration (μM)	Outcome
*Rac GTPases: Rac1‐3*								
EHT1864	40–250 nM	Unknown (none?)	100	≥ 50% inhibition of NA, PE, MTX, EFS, ET1, U46619	100	Complete inhibition NA, PE, MTX, EFS, ET1, U46619	100	(Nearly) complete inhibition CCH, MCH
					10	Nearly complete inhibition PE, EFS	10	
					1	No effect		
NSC23766	50 μM	Muscarinic antagonism	100	≥ 50% inhibition of NA, PE, MTX, EFS, U46619; no effect ET1	100	Antagonism NA, PE, MTX; full inhibition EFS, 50% U46619, no effect ET1	100	Antagonism CCH, MCH
					10	Antagonism PE	10	Inconsistent effects
					1	Slight antagonism PE		
*Cytohesin GEFs*								
SecinH3	2.4–5.6 μM cytohesins	≥ 65 μM yGea2‐S7, hEFA6‐S7; 30 μM inhibits Arf6	30	20%–50% inhibition of EFS, NA, PE, ET1, U46619	30	No effects on NA, PE, EFS	30	Inhibition CCH, MCH
							10	Inconsistent effects

*Note:* Shown are affinities and IC_50_ values for inhibition of activities (see main text for details and references), and concentrations applied to prostate tissues in previous studies and to renal interlobar arteries in this study and main outcomes on effects in contractions, together with main outcomes in contraction experiments.

Abbreviations: CCH, carbachol; EFS, electric field stimulation; ET1, endothelin‐1; MCH, methacholine; MTX, methoxamine; NA, noradrenaline; PE, phenylephrine.

**TABLE 2:** Kinase inhibitors used in this study.

	IC_50_ biochemical assays		Prostate tissues		Renal interlobar arteries	
	Target	Off‐target	Applied concentration (μM)	Outcome	Applied concentration (μM)	Outcome
*LIMK1, ‐2*						
SR7826	43 nM (LIMK1)	5.5 μM ROCK1, 6.6 μM ROCK2	1 and 0.5	Partial inhibition (≤ 50%) of NA, PE, MTX, U46619 with 1 μM; no effect of 1 μM on ET1; ≤ 50% inhibition of EFS with 0.5 μM	1	Partial inhibitions (< 50%) NA, PE, MTX, EFS, ET1, U46619
LIMKi3	7–8 nM (LIMK1/2)	Unknown	1 and 0.5	Partial inhibition (≤ 50%) of NA, PE, MTX, U46619 with 1 μM; no effect of 1 μM on ET1; no effect of 0.5 μM on EFS	1	No inhibition NA, PE, EFS
*GRK2, ‐3*						
CMPD101	32–290 nM	50% inhibition with 1 μM: PRK2, SGK1, RSK1, MSK1, S6K1	50 and 5	≥ 50% inhibition of NA, PE, EFS, ET1, U46619 with 50 μM; small inhibitions of EFS with 5 μM	50	Partial inhibition PE, MTX, U46619; no effect NA, ET1, EFS
*PAK1‐4*						
FRAX486	8–55 nM PAK1‐3, 779 nM PAK4	Unknown	30 and 10	≥ 50% inhibition of EFS, ET1, U46619 with 30 μM, no inhibition of NA, PE with 30 μM; 10 μM without effect on EFS, ET1	30	No inhibition of NA, PE, EFS
*ILK*						
Cpd22	0.6 μM	Inhibition with 5 μM: p70S6K > 50%, Abl and CDK1 27%, 17 others ≤ 10%	3	≥ 50% inhibition of EFS, U46619; no or smallest inhibition of NA, PE, MTX	3	No inhibition of NA, PE, EFS (but rather increases)

*Note:* Shown are affinities and IC_50_ values for inhibition of activities (see main text for details and references), and concentrations applied to prostate tissues in previous studies and to renal interlobar arteries in this study and main outcomes on effects in contractions, together with main outcomes in contraction experiments.

Abbreviations: EFS, electric field stimulation; ET1, endothelin‐1; MTX, methoxamine; NA, noradrenaline; PE, phenylephrine.

### 2.6 Statistical Analyses

Data are presented as means with standard deviation (SD) in concentration and frequency response curves, and as single values from each single experiment for *E*_max_, EC_50_, and EF_50_ values together with means in scatter plots. In the text, *E*_max_ and EC_50_ values are reported as means together with 95% confidence intervals (CIs). Calculation of 95% CIs and statistical analyses were performed using GraphPad Prism 6. Comparison of whole curves was performed by two‐way analysis of variance (ANOVA), as previously described [17]. Post hoc analyses for multiple comparisons at single agonist concentrations or frequencies were not performed, as this has been discouraged by the “GraphPad Statistics Guide” (GraphPad Software Inc., San Diego, CA, USA). *E*_max_, EC_50_, and EF_50_ values were compared by a paired Student's *t*‐test. *p* values < 0.05 were considered significant. Interpretation and discussion of results were based on effect sizes and their possible relevance instead of *p* values. In line with recent recommendations [37], *p* values were used sparingly, so that *p* values ≥ 0.05 have not been indicated and no *p* values are reported in the text. The present study and analyses are exploratory in nature and design, as important features of a purely hypothesis‐testing study were lacking including a clearly defined study plan without deviations, blinding, or biometric calculation of group sizes [37]. Therefore, but also because our group sizes did not allow valid testing of our data from curve fitting for normality distribution and as a non‐parametric alternative is not available for two‐way ANOVA, the *p* values reported here need to be considered descriptive, not hypothesis‐testing [37]. Normality within concentration and frequency response curves was assessed by testing the residuals of non‐linear regression fits by the Shapiro–Wilk test, separately for each control and test group. The data structure, involving three variables and repeated measurements, generated a sufficient number of residuals to allow normality testing despite small group sizes. Although test results suggested non‐normal distributions in at least one of the two groups in most of the data sets (see Table S1), 2‐way ANOVA was used due to lack of non‐parametric alternatives and its robustness against moderate violations of normality in small, paired samples [18]. Multiple post hoc comparisons, at single agonist concentrations or frequencies across groups have been previously discouraged [17]. Pre‐planned group sizes of five independent experiments were maintained for each series, whereas originally planned series on the effects of LIMKi3 with methoxamine, endothelin, and U46619 were not performed, since no inhibition with these substances was observed in experiments with noradrenaline, phenylephrine, and EFS.

### 2.7 Nomenclature of Targets and Ligands

Key protein targets and ligands in this article are hyperlinked to corresponding entries in http://www.guidetopharmacology.org, the common portal for data from the IUPHAR/BPS Guide to PHARMACOLOGY [38], and are permanently archived in the Concise Guide to PHARMACOLOGY 2023/24 [20, 39, 40].

## 3 Results

### 3.1 EHT1864

**FIGURE 1:**
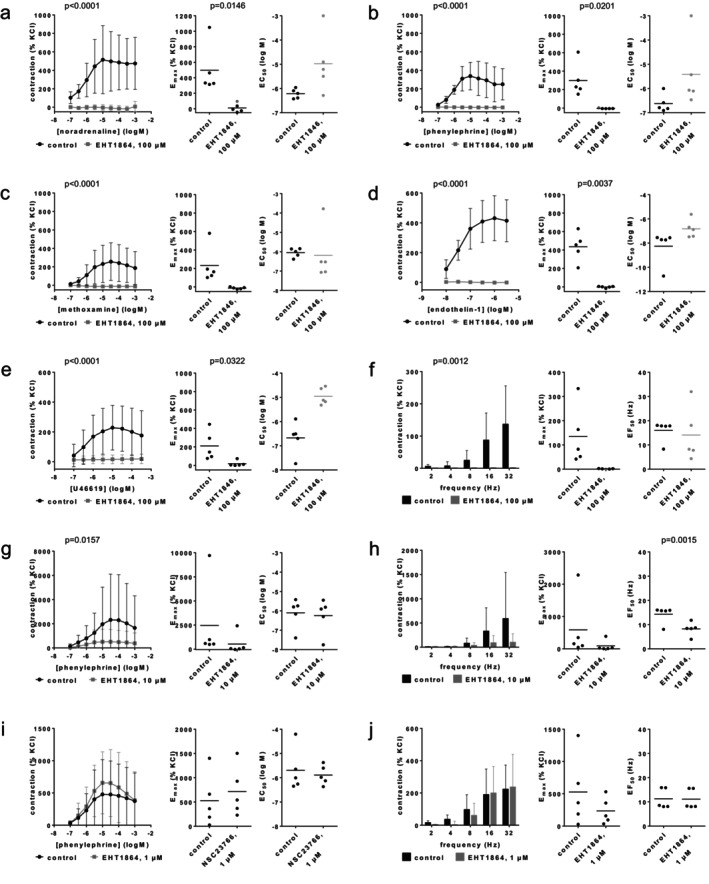
Effects of EHT1864 on agonist‐ and EFS‐induced contractions of renal, interlobar arteries. Contractions were induced 30 min after addition of solvent (deionized water) to controls or 100 μM EHT1864 (a–f), 10 μM EHT1864 (g, h) or 1 μM EHT1864 (i, j) by noradrenaline (a), phenylephrine (b, g, i), methoxamine (c), endothelin‐1 (d), U46619 (e) or EFS (f, h, j). Data are obtained from *n* = 5 independent experiments per panel, performed with arteries from *n* = 5 animals, with each single experiment including a control and EHT1864 group with tissues from the same animal, resulting in paired samples. Shown are means ± SD from all experiments in concentration and frequency response curves together with *p* values from two‐way ANOVA for whole groups, and all single *E*_max_, EC_50_, and EF_50_ values from all experiments (calculated by curve fitting) together with *p* values from Student's *t*‐test. *p* values ≥ 0.05 are not shown. *E*_max_ values marked gray represent maximum tensions in concentration or frequency response curves, as curve fitting was not possible in these experiments, and EC_50_ values of −3 or an EF_50_ of 32 Hz have been assumed for noradrenaline, phenylephrine or EFS in these experiments. While curve fitting was technically still possible in most experiments, despite nearly complete inhibition, the validity of EC_50_ and EF_50_ values with EHT1864 is limited in (a–f), so that these are marked in gray.

Contractions by noradrenaline, phenylephrine, methoxamine, endothelin‐1, U46619, and EFS in renal interlobar arteries were virtually completely inhibited by 100 μM EHT1864 (Figure 1). Full inhibitions occurred with each agonist concentration, and at each applied frequency (Figure 1). Despite apparently complete inhibition, curve fitting was technically possible in the EHT1864 groups. The resulting *E*_max_ values fully reflect the inhibitions seen in the curves, but the resulting EC_50_ values have no meaningful validity. *E*_max_ values amounted to 498% [107–889] of KCl‐induced contractions in controls and 11% [−59 to 80] with 100 μM EHT1864 for noradrenaline (Figure 1a), 299% [76–523] in controls and −4% [−9 to 1] with 100 μM EHT1864 for phenylephrine (Figure 1b), 233% [−13 to 478] in controls and −13% [−26 to 1] with 100 μM EHT1864 for methoxamine (Figure 1c), 435% [242–628] in controls and −1% [−13 to 10] with 100 μM EHT1864 for endothelin‐1 (Figure 1d), 211% [16–405] in controls and 18% [−20 to 56] with 100 μM EHT1864 for U46619 (Figure 1e), and 135% [−15 to 284] for controls and 1% [0–3] with 100 μM EHT1864 for EFS (Figure 1f).

The effects of lower concentrations were tested on phenylephrine‐ and EFS‐induced contractions of renal interlobar arteries. At 10 μM, EHT1864 still inhibited phenylephrine‐ and EFS‐induced contractions (Figure 1g,h), while 1 μM was mostly ineffective (Figure 1i,j). Phenylephrine‐ and EFS‐induced contractions were again strongly, though not completely inhibited at 10 μM (Figure 1g,h). *E*_max_ values amounted to 2459% [−2579 to 7497] of KCl‐induced contractions in controls and 522% [−799 to 1843] with 10 μM EHT1864 for phenylephrine, to 587% [−601 to 1775] in controls and 92% [−115 to 298] with 10 μM EHT1864 for EFS, to 528% [−145 to 1201] in controls and 714% [77–1351] with 1 μM EHT1864 for phenylephrine, and to 528% [−145 to 1201] in controls and 235% [−19 to 490] with 1 μM EHT1864 (Figure 1g–j). EC_50_ values for phenylephrine or EF_50_ values were not changed by 10 μM or 1 μM EHT1864, apart from a decrease in EF_50_ values by 10 μM (Figure 1h).

**FIGURE 2:**
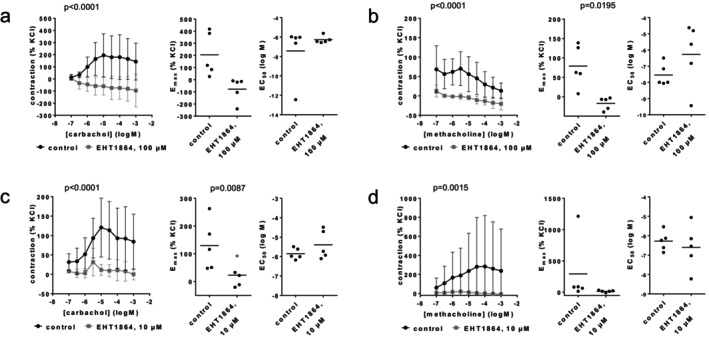
Effects of EHT1864 on cholinergic contractions of coronary arteries. Contractions were induced 30 min after addition of solvent (deionized water) to controls or 100 μM EHT1864 (a, b) or 10 μM EHT1864 (c, d) by carbachol (a, c) or methacholine (b, d). Data are obtained from *n* = 5 independent experiments per panel, performed with arteries from *n* = 5 animals, with each single experiment including a control and EHT1864 group with tissues from the same animal, resulting in paired samples. Shown are means ± SD from all experiments in concentration response curves together with *p* values from two‐way ANOVA for whole groups, and all single *E*_max_ and EC_50_ values from all experiments (calculated by curve fitting) together with *p* values from Student's *t*‐test. *p* values ≥ 0.05 are not shown. An *E*_max_ value marked gray represents the maximum tension in concentration response curves, as the value from curve fitting was implausible.

In coronary arteries, 100 μM EHT1864 completely inhibited contractions by carbachol and methacholine (Figure 2). Full inhibitions occurred with each agonist concentration and were reflected by *E*_max_ values calculated by curve fitting. *E*_max_ values amounted to 206% [−18 to 430] of KCl‐induced contractions in controls and −77% [−200 to 46] with 100 μM EHT1864 for carbachol (Figure 2a), and to 79% [12–146] in controls and −17% [−38 to 3] with 100 μM EHT1864 for methoxamine (Figure 2b). Changes in EC_50_ values were inconsistent for carbachol and methacholine (Figure 2a,b), and have no meaningful validity, as contractions were completely inhibited. Similarly, contractions were completely or nearly completely inhibited by 10 μM EHT1864. *E*_max_ values amounted to 130% [18–242] in controls and 23% [−33 to 78] with 10 μM EHT1864 for carbachol (Figure 2c), and 292% [−348 to 933] in controls and 15% [−4 to 34] with 10 μM EHT1864 for methacholine (Figure 2d). EC_50_ values were similar between control and EHT1864 groups (Figure 2c,d).

### 3.2 NSC23766

**FIGURE 3:**
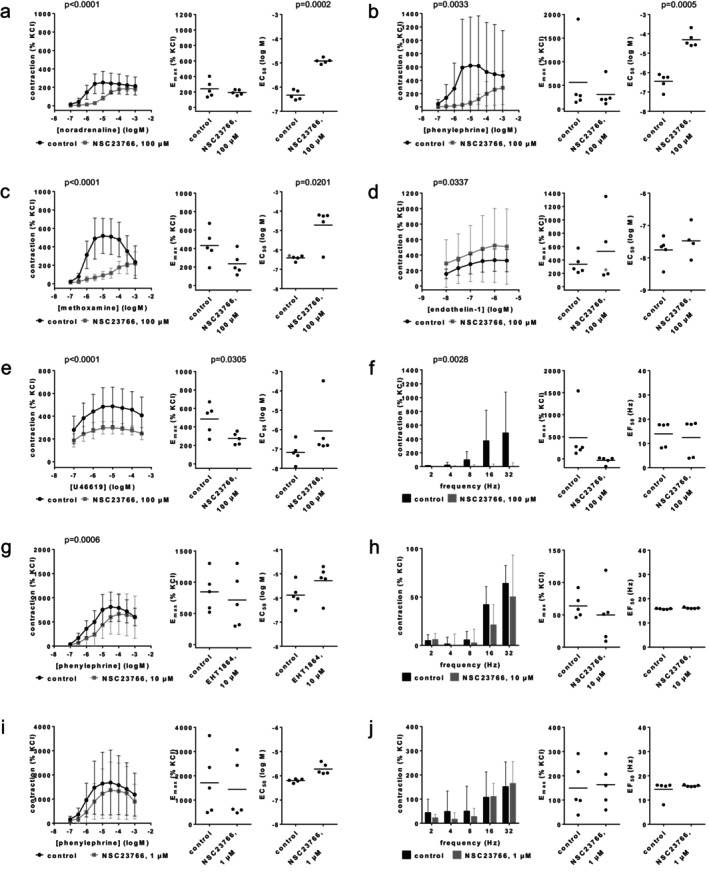
Effects of NSC23766 (100 μM) on agonist‐ and EFS‐induced contractions of renal, interlobar arteries. Contractions were induced 30 min after addition of solvent (deionized water) to controls or 100 μM NSC23766 (a–f), 10 μM NSC23766 (g, h) or 1 μM NSC23766 (i, j) by noradrenaline (a), phenylephrine (b, g, i), methoxamine (c), endothelin‐1 (d), U46619 (e) or EFS (f, h, j). Data are obtained from *n* = 5 independent experiments per panel, performed with arteries from *n* = 5 animals, with each single experiment including a control and NSC23766 group with tissues from the same animal, resulting in paired samples. Shown are means ± SD from all experiments in concentration and frequency response curves together with *p* values from two‐way ANOVA for whole groups, and all single *E*_max_, EC_50_, and EF_50_ values from all experiments (calculated by curve fitting) together with *p* values from Student's *t*‐test. *p* values ≥ 0.05 are not shown. An *E*_max_ value marked in gray represents the maximum tension in the concentration response curves, as curve fitting was not possible in this experiment, while no EC_50_ value for endothelin‐1 could be estimated for this experiment.

Concentration response curves for noradrenaline, phenylephrine, and methoxamine in renal interlobar arteries were right‐shifted by 100 μM NSC23766 (Figure 3a–c). Right shifts were accompanied by full recovery of noradrenaline‐induced contractions, and partial recovery of phenylephrine‐ and methoxamine‐induced contractions at the applied agonist concentrations (0.1–100 μM), together with increases in EC_50_ values by more than one order of magnitude for each of the three agonists. EC_50_ values (log M) were −6.3 [−6.6 to −6.1] in controls and −4.9 [−5.1 to −4.8] with 100 μM NSC23766 for noradrenaline (Figure 3a), −6.5 [−7 to −5.9] in controls and −4.3 [−4.8 to −3.8] with 100 μM NSC23766 for phenylephrine (Figure 3b), and −6.4 [−6.6 to −6.2] in controls and −4.7 [−5.9 to −3.6] with 100 μM NSC23766 for methoxamine (Figure 3c). *E*_max_ values amounted to 242% [106–378] of KCl‐induced contraction in controls and 195% [153–237] with 100 μM NSC23766 for noradrenaline (Figure 3a), 564% [−367 to 1497] in controls and 309% [−31 to 648] with 100 μM NSC23766 for phenylephrine (Figure 3b), and 433% [213–653] in controls and 236% [85–386] with 100 μM NSC23766 for methoxamine (Figure 3c). Contractions by endothelin‐1 were not inhibited by NSC23766, and right shifts of concentration response curves did not occur with NSC23766 (Figure 3d). *E*_max_ values for endothelin‐1 amounted to 336% [152–521] in controls and 530% [−94 to 1153] with 100 μM NSC23766 (Figure 3d). EC_50_ values (log M) for endothelin‐1 were −7.6 [−7.9 to −7.3] in controls and −7.5 [−8.3 to −6.7] with 100 μM NSC23766 (Figure 3d). Contractions by U46619 were inhibited by NSC23766, reflected by reduced *E*_max_ values but without right shifts of concentration response curves (Figure 3e). *E*_max_ values for U46619 amounted to 485% [281–689] in controls and 273% [194–353] with 100 μM NSC23766 (Figure 3e). EC_50_ values (log M) for U46619 were −7.2 [−7.9 to −6.5] in controls and −6.1 [−7.9 to −4.3] with 100 μM NSC23766 (Figure 2e). EFS‐induced contractions were virtually completely inhibited at each frequency, though calculation of *E*_max_ values was technically still possible (Figure 3f). *E*_max_ values for EFS‐induced contractions amounted to 482% [−258 to 1220] in controls and −35% [−134 to 64] with 100 μM NSC23766 (Figure 3f).

Apparent pA_2_ values for NSC23766 were calculated for series with α_1_‐adrenergic agonists and 100 μM NSC23766, which was possible for each experiment in these series. The average apparent pA_2_ was 5.2 [3.7–6.8] with noradrenaline, 5.2 [3.4–7] with phenylephrine and 3.9 [2.3–5.4] with methoxamine, suggesting affinities of 47, 27, and 461 μM, respectively.

The effects of lower concentrations were tested on phenylephrine‐ and EFS‐induced contractions of renal interlobar arteries. While *E*_max_ values or contractions in frequency response curves were hardly or not affected by 10 μM or 1 μM NSC23766, both concentrations still right shifted concentration response curves and increased the EC_50_ values for phenylephrine (Figure 3g–j). EC_50_ values for phenylephrine (log M) mounted to −5.9 [−6.5 to −5.3] in controls and −5.3 [−6.1 to −4.5] with 10 μM NSC23766 (Figure 3g), and to −6.2 [−6.3 to −6.1] in controls and −5.7 [−6 to −5.4] with 1 μM NSC23766 (Figure 3j).

**FIGURE 4:**
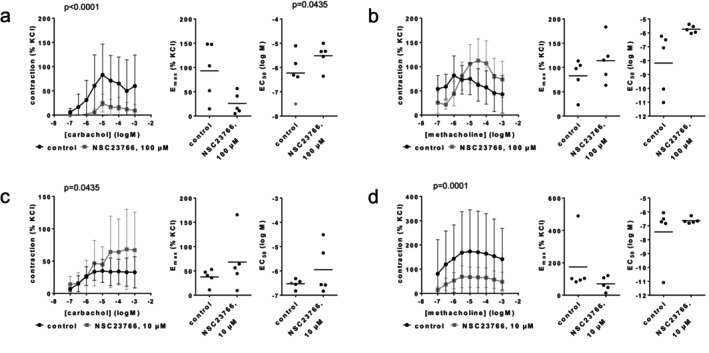
Effects of NSC23766 on cholinergic contractions of coronary arteries. Contractions were induced 30 min after addition of solvent (deionized water) to controls or 100 μM NSC23766 (a, b) or 10 μM NSC23766 (c, d) by carbachol (a, c) or methacholine (b, d). Data are obtained from *n* = 5 independent experiments per panel, performed with arteries from *n* = 5 animals, with each single experiment including a control and NSC23766 group with tissues from the same animal, resulting in paired samples. Shown are means ± SD from all experiments in concentration response curves together with *p* values from two‐way ANOVA for whole groups, and all single *E*_max_ and EC_50_ values from all experiments (calculated by curve fitting) together with *p* values from Student's *t*‐test. *p* values ≥ 0.05 are not shown. EC_50_ values marked gray represent estimations from concentration response curves, as values from curve fitting were implausible.

In coronary arteries, 100 μM NSC23766 increased the EC_50_ values for carbachol and methacholine (Figure 4a,b). Increased EC_50_ values for carbachol were paralleled by decreases in *E*_max_ values and by inhibitions at all agonist concentrations in concentration response curves (Figure 4a). Increased EC_50_ values for methacholine were paralleled by right shifts of concentration response curves, with recovery at high methacholine concentrations (Figure 4b). EC_50_ (log M) values amounted to −6.3 [−7.3 to −5.1] in controls and −5.5 [−6.1 to −4.9] with 100 μM NSC23766 for carbachol, and to −8.2 [−10.9 to −5.4] in controls and −5.7 [−6.1 to −5.4] with 100 μM NSC23766 for methacholine (Figure 4a,b). *E*_max_ values amounted to 93% [20–167] of KCl‐induced contraction in controls and 26% [−2 to 53] with 100 μM NSC23766 for carbachol, and 83% [38–128] in controls and 114% [58–171] with 100 μM NSC23766 for methacholine (Figure 4a,b). At 10 μM NSC23766, contractions with carbachol and methacholine were not consistently affected (Figure 4c,d). EC_50_ values amounted to −6.5 [−6.8 to −6.3] in controls and −5.9 [−7.2 to −4.7] with 10 μM NSC23766 for carbachol, and to −7.4 [−10 to −4.9] in controls and −6.6 [−6.9 to −6.4] with 10 μM NSC23766 for methacholine (Figure 4c,d). *E*_max_ values amounted to 38% [17, 18, 19, 20, 21, 22, 23, 24, 25, 26, 27, 28, 29, 30, 31, 32, 33, 34, 35, 36, 37, 38, 39, 40, 41, 42, 43, 44, 45, 46, 47, 48, 49, 50, 51, 52, 53, 54, 55, 56, 57, 58] in controls and 68% [−4 to 141] with 10 μM NSC23766 for carbachol, and to 175% [−44 to 393] in controls and 70% [15–129] with 10 μM NSC23766 for methacholine (Figure 4c,d).

### 3.3 SecinH3

**FIGURE 5:**
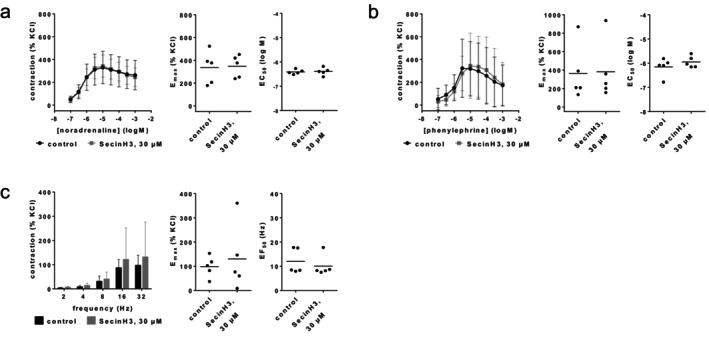
Effects of SecinH3 (30 μM) on agonist‐ and EFS‐induced contractions of renal, interlobar arteries. Contractions were induced by noradrenaline (a), phenylephrine (b) or EFS (c), 30 min after the addition of solvent (DMSO) to controls or SecinH3 (30 μM). Data are obtained from *n* = 5 independent experiments per panel, performed with arteries from *n* = 5 animals, with each single experiment including a control and SecinH3 group with tissues from the same animal, resulting in paired samples. Shown are means ± SD from all experiments in concentration and frequency response curves, and all single *E*_max_, EC_50_, and EF_50_ values from all experiments (calculated by curve fitting).

Contractions by noradrenaline, phenylephrine or EFS were not changed with SecinH3 (30 μM) (Figure 5). *E*_max_ values amounted to 336% [158–513] of KCl‐induced contractions in controls and 348% [228–469] with SecinH3 for noradrenaline (Figure 5a), 363% [−8 to 733] in controls and 382% [−15 to 778] with SecinH3 for phenylephrine (Figure 5b), and 99% [45–152] in controls and 130% [−41 to 302] with SecinH3 for EFS (Figure 5c). EC_50_ values for both agonists and EF_50_ values for EFS remained unchanged by SecinH3 (Figure 5).

**FIGURE 6:**
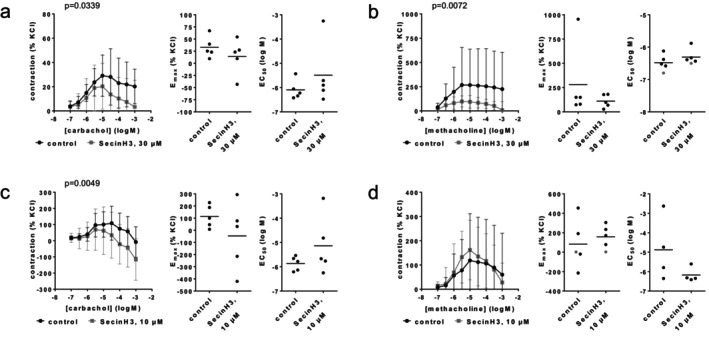
Effects of SecinH3 on cholinergic contractions of coronary arteries. Contractions were induced 30 min after addition of solvent (DMSO) to controls or 30 μM SecinH3 (a, b) or 10 μM SecinH3 (c, d) by carbachol (a, c) or methacholine (b, d). Data are obtained from *n* = 5 independent experiments per panel, performed with arteries from *n* = 5 animals, with each single experiment including a control and SecinH3 group with tissues from the same animal, resulting in paired samples. Shown are means ± SD from all experiments in concentration response curves together with *p* values from two‐way ANOVA for whole groups, and all single *E*_max_ and EC_50_ values from all experiments (calculated by curve fitting) analyzed by Student's *t*‐test. *p* values ≥ 0.05 are not shown. *E*_max_ values marked gray represent maximum tensions in concentration response curves, as curve fitting was not possible in these experiments, and EC_50_ values marked in gray have been estimated from concentration response curves in these experiments.

In coronary arteries, 30 μM SecinH3 inhibited contractions by carbachol and methacholine (Figure 6a,b). Inhibitions were obvious in concentration response curves at carbachol concentrations from 10 μM to 1 mM, and at methacholine concentrations from 1 μM to 1 mM, and were reflected by reduced *E*_max_ values. *E*_max_ values amounted to 33% of KCl‐induced contractions [6, 7, 8, 9, 10, 11, 12, 13, 14, 15, 16, 17, 18, 19, 20, 21, 22, 23, 24, 25, 26, 27, 28, 29, 30, 31, 32, 33, 34, 35, 36, 37, 38, 39, 40, 41, 42, 43, 44, 45, 46, 47, 48, 49, 50, 51, 52, 53, 54, 55, 56, 57, 58, 59, 60] in controls and 14% [−30 to 59] with 30 μM SecinH3 for carbachol, and to 281% [−188 to 751] in controls and 111% [27–195] with 30 μM SecinH3 for methacholine (Figure 6a,b). At 10 μM SecinH3, contractions by carbachol or methacholine were not consistently changed (Figure 6c,d). EC_50_ values for carbachol or methacholine were not consistently changed at 10 μM or 30 μM SecinH3 (Figure 6c,d).

### 3.4 SR7826

**FIGURE 7:**
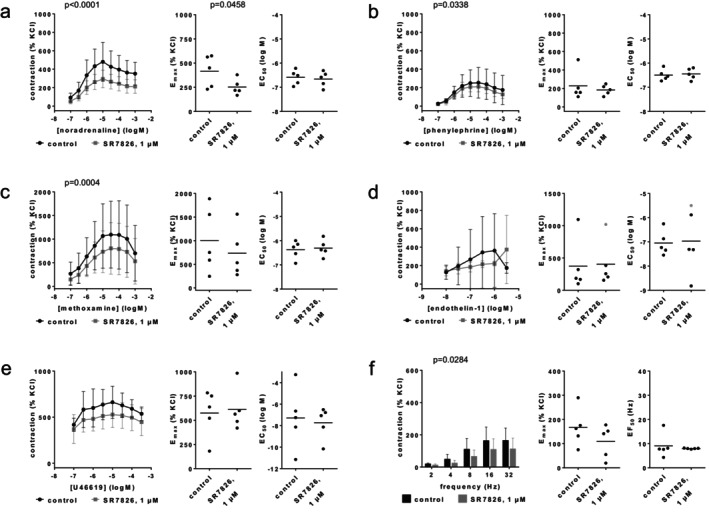
Effects of SR7826 (1 μM) on agonist‐ and EFS‐induced contractions of renal, interlobar arteries. Contractions were induced by noradrenaline (a), phenylephrine (b), methoxamine (c), endothelin‐1 (d), U46619 (e) or EFS (f), 30 min after addition of solvent (DMSO) to controls or SR7826 (1 μM). Data are obtained from *n* = 5 independent experiments per panel, performed with arteries from *n* = 5 animals, with each single experiment including a control and SR7826 group with tissues from the same animal, resulting in paired samples. Shown are means ± SD from all experiments in concentration and frequency response curves together with *p* values from two‐way ANOVA for whole groups, and all single *E*_max_, EC_50_, and EF_50_ values from all experiments (calculated by curve fitting) together with *p* values from Student's *t*‐test. *p* values ≥ 0.05 are not shown. An *E*_max_ value marked in gray represents the maximum tension in the concentration response curves, as curve fitting was not possible in this experiment, while no EC_50_ value for endothelin‐1 could be estimated for this experiment.

Contractions by noradrenaline, phenylephrine, methoxamine, endothelin‐1, U46619 and EFS were partly inhibited by SR7826 (1 μM) (Figure 7). For α_1_‐adrenergic agonists and EFS, but not for endothelin‐1 or U46619, the extent of inhibition was reflected in the *E*_max_ values. *E*_max_ values amounted to 415% [212–619] of KCl‐induced contractions in controls and 252% [151–364] with SR7826 for noradrenaline (Figure 7a), 228% [27–429] in controls and 183% [115–251] with SR7826 for phenylephrine (Figure 7b), 1005% [150–1860] in controls and 735% [83–1386] with SR7826 for methoxamine (Figure 7c), 375% [−136 to 886] in controls and 404% [−36 to 845] with SR7826 for endothelin‐1 (Figure 7d), 575% [273–877] in controls and 612% [338–885] with SR7826 for U46619 (Figure 7e), and 167% [69–265] for controls and 109% [25–193] with SR7826 for EFS (Figure 7f). EC_50_ values of all agonists and EF_50_ values for EFS remained unchanged by SR7826 (Figure 7).

### 3.5 LIMKi3

**FIGURE 8:**
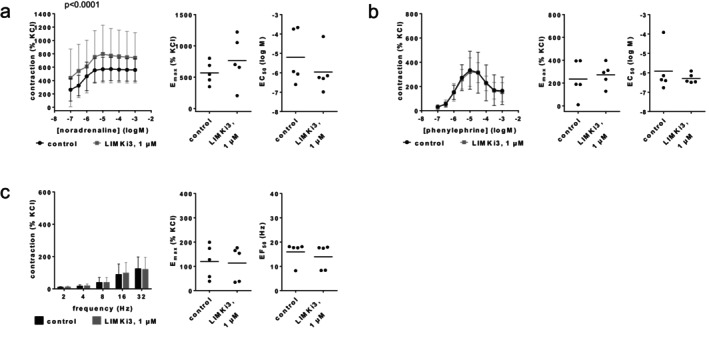
Effects of LIMKi3 (1 μM) on agonist‐ and EFS‐induced contractions of renal, interlobar arteries. Contractions were induced by noradrenaline (a), phenylephrine (b) or EFS (c), 30 min after addition of solvent (DMSO) to controls or LIMKi3 (1 μM). Data are obtained from *n* = 5 independent experiments per panel, performed with arteries from *n* = 5 animals, with each single experiment including a control and LIMKi3 group with tissues from the same animal, resulting in paired samples. Shown are means ± SD from all experiments in concentration and frequency response curves together with *p* values from two‐way ANOVA for whole groups, and all single *E*_max_, EC_50_, and EF_50_ values from all experiments (calculated by curve fitting). *p* values ≥ 0.05 are not shown. *E*_max_ and EC_50_ values marked in gray represent the maximum tension in the concentration response curves, or the highest applied endothelin‐1 concentration, as curve fitting was not possible in this experiment.

Contractions by noradrenaline, phenylephrine or EFS were not reduced with LIMKi3 (1 μM) (Figure 8). *E*_max_ values amounted to 568% [344–792] of KCl‐induced contractions in controls and 767% [279–1256] with LIMKi3 for noradrenaline (Figure 8a), 235% [33–437] in controls and 273% [149–396] with LIMKi3 for phenylephrine (Figure 8b), and 119% [32–207] in controls and 114% [25–202] with LIMKi3 for EFS (Figure 8c). EC_50_ values for both agonists and EF_50_ values for EFS remained unchanged by LIMKi3 (Figure 8).

### 3.6 CMPD101

**FIGURE 9:**
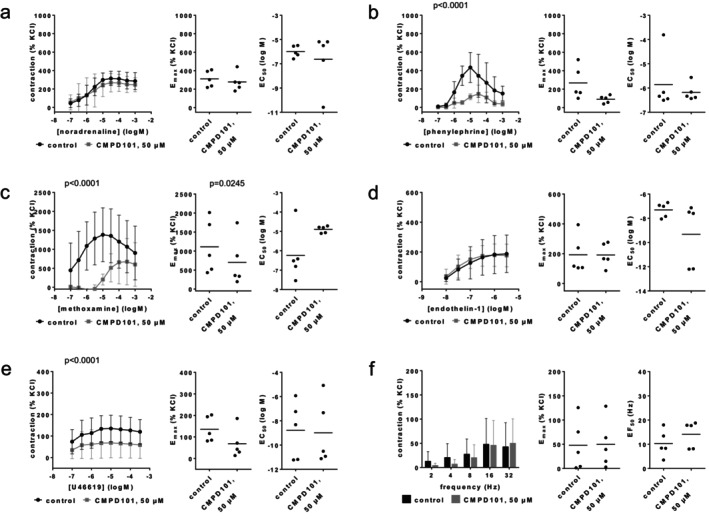
Effects of CMPD101 (50 μM) on agonist‐ and EFS‐induced contractions of renal, interlobar arteries. Contractions were induced by noradrenaline (a), phenylephrine (b), methoxamine (c), endothelin‐1 (d), U46619 (e) or EFS (f), 30 min after the addition of solvent (ethanol) to controls or CMPD (50 μM). Data are obtained from *n* = 5 independent experiments per panel, performed with arteries from *n* = 5 animals, with each single experiment including a control and CMPD101 group with tissues from the same animal, resulting in paired samples. Shown are means ± SD from all experiments in concentration and frequency response curves together with *p* values from two‐way ANOVA for whole groups, and all single *E*_max_, EC_50_, and EF_50_ values from all experiments (calculated by curve fitting) together with *p* values from Student's *t*‐test. *p* values ≥ 0.05 are not shown.

Agonist‐ and EFS‐induced contractions were differentially affected by CMPD101 (50 μM) (Figure 9). Contractions by noradrenaline, endothelin‐1 and EFS were unchanged with CMPD101. Maximum phenylephrine‐ and U46619‐induced contractions were reduced with CMPD101. Concentration response curves for methoxamine were right shifted with CMPD101, together with increases in EC_50_ values and decreases in *E*_max_ values for methoxamine. *E*_max_ values amounted to 312% [203–421] of KCl‐induced contractions in controls and 278% [154–402] with CMPD101 for noradrenaline (Figure 9a), 267% [48–487] in controls and 90% [39–141] with CMPD101 for phenylephrine (Figure 9b), 1110% [230–1991] in controls and 701% [−90 to 1493] with CMPD101 for methoxamine (Figure 9c), 192% [36–349] in controls and 191% [93–290] with CMPD101 for endothelin‐1 (Figure 9d), 136% [63–209] in controls and 69% [−16 to 154] with CMPD101 for U46619 (Figure 9e), and 48% [−17 to 113] for controls and 50% [−12 to 112] with CMPD101 for EFS (Figure 9f). EC_50_ values (log M) were −6.2 [−7.9 to −4.5] for controls and −4.9 [−5.1 to −4.7] with CMPD101 for methoxamine (Figure 9c), while EC_50_ values for other agonists and EF_50_ values for EFS did not change.

### 3.7 FRAX486

**FIGURE 10:**
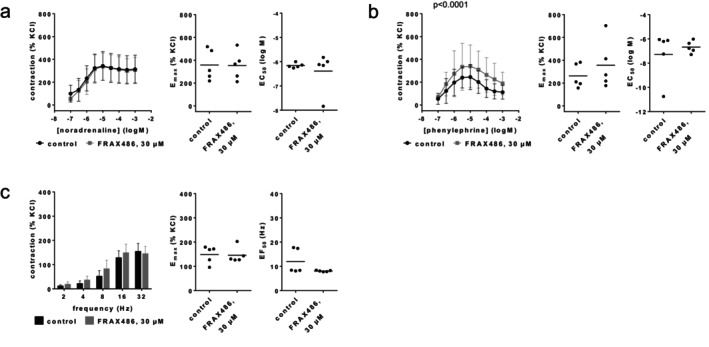
Effects of FRAX486 (30 μM) on agonist‐ and EFS‐induced contractions of renal, interlobar arteries. Contractions were induced by noradrenaline (a), phenylephrine (b) or EFS (c), 30 min after the addition of solvent (DMSO) to controls or FRAX486 (30 μM). Data are obtained from *n* = 5 independent experiments per panel, performed with arteries from *n* = 5 animals, with each single experiment including a control and FRAX486 group with tissues from the same animal, resulting in paired samples. Shown are means ± SD from all experiments in concentration and frequency response curves together with *p* values from two‐way ANOVA for whole groups, and all single *E*_max_, EC_50_, and EF_50_ values from all experiments (calculated by curve fitting). *p* values ≥ 0.05 are not shown.

Contractions by noradrenaline, phenylephrine or EFS were not reduced with FRAX486 (30 μM) (Figure 10). *E*_max_ values amounted to 359% [191–527] of KCl‐induced contractions in controls and 354% [199–508] with FRAX486 for noradrenaline (Figure 10a), 263% [133–393] in controls and 357% [92–621] with FRAX486 for phenylephrine (Figure 10b), and 149% [104–193] in controls and 146% [106–186] with FRAX486 for EFS (Figure 10c). EC_50_ values for both agonists and EF_50_ values for EFS remained unchanged by FRAX486 (Figure 10).

### 3.8 Cpd22

**FIGURE 11:**
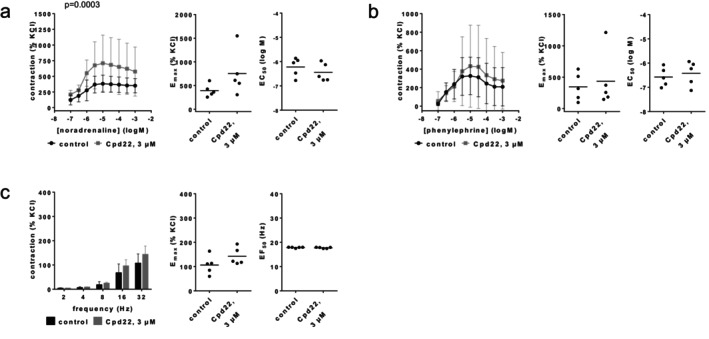
Effects of Cpd22 (3 μM) on agonist‐ and EFS‐induced contractions of renal, interlobar arteries. Contractions were induced by noradrenaline (a), phenylephrine (b) or EFS (c), 30 min after the addition of solvent (DMSO) to controls or Cpd22 (3 μM). Data are obtained from *n* = 5 independent experiments per panel, performed with arteries from *n* = 5 animals, with each single experiment including a control and Cpd22 group with tissues from the same animal, resulting in paired samples. Shown are means ± SD from all experiments in concentration and frequency response curves together with *p* values from two‐way ANOVA for whole groups, and all single *E*_max_, EC_50_, and EF_50_ values from all experiments (calculated by curve fitting). *p* values ≥ 0.05 are not shown.

Contractions by noradrenaline, phenylephrine or EFS were not reduced with Cpd22 (3 μM) (Figure 11). *E*_max_ values amounted to 395% [231–558] of KCl‐induced contractions in controls and 753% [165–1340] with Cpd22 for noradrenaline (Figure 11a), 346% [68–624] in controls and 434% [−117 to 986] with Cpd22 for phenylephrine (Figure 11b), and 107% [59–155] in controls and 143% [99–187] with Cpd22 for EFS (Figure 11c). EC_50_ values for both agonists and EF_50_ values for EFS remained unchanged by Cpd22 (Figure 11).

## 4 Discussion

The present study aims at a first preclinical investigation of the vasoactive effects of compounds recently described to inhibit prostatic smooth muscle contractions (Tables 1 and 2). Adverse cardiovascular events are critical in the treatment of voiding symptoms suggestive of BPH, so the potential to induce hypotension needs to be considered in the development of new drugs. Concentrations of the test compounds applied in the initial experiments of our study (i.e., using the highest examined concentrations) were the concentrations used in previous studies using human prostate tissues, to ensure comparability between both tissue types. These concentrations may partly exceed the IC_50_ values known from biochemical assays and might have limited physiological relevance, but plasma levels are not known and the absence of effects with these concentrations may suggest lacking hypotensive effects with these or lower concentrations in vivo. The vessel types used here are associated with renal function or myocardial perfusion. Accordingly, firm conclusions regarding hypotensive effects remain subject to further investigations in other vessel types and human arteries, and to hemodynamic measurements in vivo. Typically for vascular smooth muscle, however, our renal arteries showed α_1_‐adrenergic, and endothelin‐1‐ and U46619‐induced contractions, while we did not include angiotensin‐II, serotonin or other important vasoconstrictors. Different from most (if not all) other vessel types, coronary arteries hardly contract adrenergically or with EFS, but typically show cholinergic contractions [17, 41].

The largest and most consistent effects occurred with EHT1864 and NSC23766. With both compounds, anticontractile effects were pronounced at 100 μM in both vessel types, still strong with 10 μM EHT1864 in both vessel types, and still visible, though weaker with 10 μM NSC23766 in renal arteries, but lacking with 1 μM EHT1864 in renal arteries or with 10 μM NSC23766 in coronary arteries. Inhibitions ≥ 50% were also observed for α_1_‐adrenergic, EFS‐ and U46619‐induced contractions with both compounds in prostate tissues, and for EHT1864 additionally with U46619 [10, 11]. Although the specificity at 100 μM may be limited, 100 μM EHT1864 or NSC23766, or 200 μM NSC23766 did not inhibit the closely related, contraction‐mediating GTPase RhoA [10, 24, 25]. EHT1864 binds to Rac1‐3 with *K*_d_ values of 40–250 nM, but clear IC_50_ values for enzyme activity have not been reported and the IC_50_ for inhibition of Rac‐mediated functions in cell cultures ranges around 5–10 μM [21, 22, 25, 42]. The IC_50_ for GEF‐induced Rac1 inhibition by NSC23766 in biochemical assays amounts to 50 μM [24], consistent with functional readouts if concentration‐dependent effects were examined [25]. NSC23766 is typically used at concentrations of 50–100 μM. Lower concentrations have rarely been studied [25, 43], and can be ineffective [25]. Off‐targets for EHT1864 and NSC23766 remain unknown [44], apart from NSC23766 binding to muscarinic receptors [26, 27]. At least in bladder smooth muscle cells, responses to both compounds are unaffected by Rac1 knockout, indicating Rac1‐independent effects [25]. A completely unspecific action contributing to anticontractile effects such as cytotoxicity can be excluded, as 100 μM NSC23766 failed to inhibit endothelin‐1‐induced contractions in both prostate and vascular tissues [10, 11].

Right shifts of concentration response curves, and increases of EC_50_ values with all three α_1_‐adrenergic agonists point to antagonism of α_1_‐adrenoceptors as a novel off‐target effect of NSC23766. Interpreting our data as α_1_‐adrenoceptor antagonism by NSC23766 is plausible, but still requires confirmation by radioligand binding assays. Antagonism of muscarinic receptors has previously been described as another off‐target effect of NSC23766. The EC_50_ for carbachol was increased by half to one order of magnitude by 100 μM NSC23766 in female and male human detrusor tissues [27]. In HEK‐293 cells transfected with M_1_, M_2_ or M_3_ receptors, 100 μM NSC23766 increased the EC_50_ for carbachol‐induced increases in intracellular calcium by 1.5–2 orders of magnitude, and the affinity for M_3_ was calculated to be about 4 μM by Schild regression [26]. In our experiments with coronary arteries, antagonism of muscarinic receptors was again reflected by increases in EC_50_ values for carbachol and methacholine at 100 μM NSC23766, while no changes in EC_50_ values were observed at 10 μM. Based on our apparent pA_2_ values, the affinity for α_1_‐adrenoceptors may appear lower than for muscarinic receptors, although our approach is only an approximation to an affinity. Features of an antagonism were still apparent using 10 and 1 μM NSC23766 in phenylephrine‐induced contractions, whereas 10 μM failed to produce signs of an antagonism of carbachol‐ or methacholine‐induced contractions in renal arteries. Though adrenoceptors and muscarinic receptors belong to different families, they are not that distally related among G protein‐coupled receptors [45, 46], and off‐target effects may occur with representatives of both families, for example, with mirabegron or other compounds [47, 48].

Based on previous and our current results, the use of Rac inhibitors in BPH treatment may be associated with cardiovascular effects. A role for Rac1 in smooth muscle contractions is increasingly emerging from knockout and silencing models [49]. Similar to the closely related GTPase RhoA, this function occurs across organs including vascular, bladder, airway, and gastrointestinal smooth muscle [49, 50]. Most in vitro studies indicated anticontractile effects of Rac inhibitors in isolated smooth muscle‐rich tissues. An exception was mesenteric arteries, where 10 μM EHT1864 inhibited phenylephrine‐induced contractions by 20% in arteries from spontaneously hypertensive rats, but not in vessels from normotensive controls [51]. In vivo, both inhibitors have been applied in rodents, using up to 5 mg/kg (i.p.) for NSC23766 [52, 53], or 10–40 mg/kg (i.p.) for EHT1864 [54, 55], but plasma concentrations are unknown. Effects of NSC23766 on mean arterial pressure (MAP) have been studied in normo‐ and hypertensive rats. A single dose of 100 pmol, by injection into the commissural nucleus of the solitary tract reduced the blood pressure by 20 mmHg in hypertensive rats, but was without hemodynamic effects in normotensive rats [56]. Hemodynamic data allowing conclusions on direct effects on vascular smooth muscle cells, analog to our organ bath experiments, are to the best of our knowledge not available for NSC23766 or EHT1864. Findings for hemodynamic effects from transgenic approaches are divergent, and do not consistently suggest hypotension by Rac1 inhibition. Smooth muscle‐specific overexpression of constitutively active Rac1 induced moderate hypertension [57]. In contrast, mice with smooth muscle‐specific Rac1 knockout developed hypertension as well, due to increased vascular resistance but not due to changes in diastolic blood pressure or heart rate, by disruption of nitric oxide‐induced relaxation in vascular smooth muscle cells [58].

In prostate tissues, agonist‐ and EFS‐induced contractions were consistently reduced by SecinH3 [12]. Here, we did not observe any inhibition in renal arteries, but observed inhibition of cholinergic contractions in coronary arteries. Effects in the prostate involved inhibition of Arf6, a downstream GTPase of cytohesin GEFS [12]. Arf6‐mediated contraction has been proposed based on observations using the Arf6 inhibitor NAV2729 in human prostate tissues [59], and has been evidenced by Arf6 knockout in cultured prostate stromal cells [60]. In renal interlobar arteries, responses to all six examined vasoconstrictors previously remained unaffected by NAV2729 [17]. Again in line with our current findings using SecinH3, however, NAV27632 reduced carbachol‐ and methacholine‐induced contractions in coronary arteries [17]. Pending further findings, the cytohesin‐2/Arf6 axis may represent an organ‐ and receptor‐selective pathway of smooth muscle contraction [61], allowing pharmacological targeting of prostate smooth muscle contraction without affecting vasocontraction.

For SR7826, our results largely agree with those from prostates, except endothelin‐1 being inhibited in renal arteries but not in the prostate [11, 13]. Whether partial inhibitions of agonist‐ and EFS‐induced contractions by SR7826 are sufficient for urodynamic or hemodynamic effects in vivo, remains to be determined. In biochemical assays, the IC_50_ of SR7826 was determined as 43 nM for LIMK1, but 5.5 and 6.5 μM for ROCK1 and ‐2 [30], so that ROCK inhibition appears unlikely in our experiments using 1 μM. Rho kinase is central in the contraction of all smooth muscle types. A role of LIMK for smooth muscle contraction can be easily derived, as it is central for actin‐dependent functions [62], but evidence from knockout or silencing in contraction models is still missing. Our observations with LIMKi3 were divergent from those with SR7826 and with prostate tissues [11, 13], possibly pointing to unknown pharmacological properties or to off‐target effects, which may predominate over specific LIMK inhibition in previous experiments with prostate tissues.

Findings with CMPD101 appear ambiguous, for renal arteries and prostate tissues [14]. Phenylephrine‐ and methoxamine‐induced contractions were markedly inhibited in concentration response curves, while noradrenaline‐ and EFS‐induced contractions remained unchanged. Marked inhibitions have been previously observed in prostate tissues with α_1_‐adrenergic agonists, endothelin‐1, U46619 and EFS, which was already surprising, as enhanced rather than inhibited contractions were expected from a GRK2/3 inhibitor [14]. Phosphorylation of G protein‐coupled receptors by GRKs results in receptor desensitization [1], so that suppressing this desensitization should amplify receptor‐induced contractions. Divergent effects on endothelin‐1‐ and U46619‐induced contractions may reflect different contractile mechanisms used by these receptors. Considering our results from arteries, possible hypotensive effects would be expected in vivo with CMPD101.

Our experiments with FRAX486 and Cpd22 yielded inconsistent results that are difficult to interpret. Unlike with prostate tissues, we observed no effects on EFS but enhanced phenylephrine‐ or noradrenaline‐induced contractions with FRAX486 and Cpd22 in renal arteries. Whether these inconsistencies reflect organ‐specific mechanisms of neurogenic contractions, different properties of α_1_‐adrenergic ligands used here, or tissue‐ and subtype‐specific properties of α_1_‐adrenoceptor populations, can not be estimated based on our data. In any case, these compounds do not seem to be candidates for further development as BPH or antihypertensive drugs. Effects of Cpd22 on MAP have been examined in rats with experimentally‐induced severe pulmonary hypertension, where treatment for 5 days by intraperitoneal injection of 20 mg/kg per day did not affect the MAP in male and female animals [63].

Inhibition of vasocontraction is central in the treatment of cardiovascular diseases, but also accounts for side effects in the treatment of voiding symptoms with α_1_‐blockers and tadalafil [3]. Orthostatic hypotension and dizziness belong to the most frequent side effects of α_1_‐blockers [1, 3]. The risk of experiencing α_1_‐blocker‐induced hypotension is highest in patients with cardiovascular comorbidity and cardiovascular co‐medication [1, 4]. Side effects of α_1_‐blockers may be additive with those of other medications, becoming most problematic in multidrug regimens [5]. Combinations of antihypertensives with α_1_‐blockers belong to the most commonly prescribed multidrug clusters in men aged ≥ 70 years [5]. In addition, improvements by available BPH drugs are limited and about one third of treated patients are non‐responders, together calling for further drugs [1].

Because SecinH3 did not inhibit vasocontractions and subject to any further, unknown limitations, its use without cardiovascular side effects might be possible in BPH. The use of Rac inhibitors would be expected to cause cardiovascular effects. A possible advantage over α_1_‐blockers may arise from inhibition of non‐adrenergic prostate smooth muscle contractions, which are insensitive to α_1_‐blockers and are suspected of causing drug‐refractory symptoms [1]. Hemodynamic side effects should be expected also with SR7826, whereas the risk for cardiovascular effects can not be consistently estimated for all of the examined kinase inhibitors. Besides cardiovascular side effects, further side effects are likely with kinase inhibitors, which may limit their use in non‐malignant diseases. Our study aimed at an assessment of potential vasomotoric effects. Any further translational step still needs assessments of safety and tolerability, of the reversibility of vasorelaxation effects, of effects in further vessel types for each of the test compounds, of hemodynamic effects and cytotoxicity. Based on its pilot character, our study did not include mechanistic investigations into why certain inhibitors selectively affect prostatic versus vascular tissue, though this will be certainly of interest in follow‐up studies.

## Author Contributions

**Guangyang Liu:** investigation, writing – review and editing, formal analysis, data curation. **Sheng Hu:** investigation, formal analysis, writing – review and editing. **Alexander Tamalunas:** investigation, writing – review and editing. **Oluwafemi Kale:** investigation, writing – review and editing. **Yajie Xu:** investigation, writing – review and editing. **Christian G. Stief:** conceptualization, methodology, writing – review and editing, supervision, resources. **Martin Hennenberg:** conceptualization, investigation, writing – original draft, methodology, formal analysis, project administration, data curation, supervision, resources.

## Ethics Statement

This study was performed using arteries obtained from domestic animals, bred and sacrificed for meat production, that is, from pig kidneys obtained from a slaughterhouse by a butcher. Consequently, no ethical approval and no approval for animal experiments were required.

## Consent

The authors have nothing to report.

## Conflicts of Interest

The authors declare no conflicts of interest.

## Supporting information


**Table S1:** *P* values from Shapiro‐Wilk W tests, for assessment of residuals of concentration response and frequency response curves for normality. *P* values ≥ 0.05 suggest normal distribution. Compare main text for limitations and details.

## Data Availability

All data that support the findings of this study are included in this published article. Raw data are available from the corresponding author upon reasonable request.

## References

[1] M. Hennenberg and M. C. Michel , “Adrenoceptors in the Lower Urinary Tract,” Handbook of Experimental Pharmacology 285 (2024): 333–367. 37455288 10.1007/164_2023_678

[2] H. Lepor , “Pathophysiology, Epidemiology, and Natural History of Benign Prostatic Hyperplasia,” Revista de Urología 6, no. Suppl 9 (2004): S3–S10. PMC1472917 16985922

[3] S. Gravas , M. Gacci , C. Gratzke , et al., “Summary Paper on the 2023 European Association of Urology Guidelines on the Management of Non‐Neurogenic Male Lower Urinary Tract Symptoms,” European Urology 84, no. 2 (2023): 207–222. 37202311 10.1016/j.eururo.2023.04.008

[4] M. Oelke , A. Bachmann , A. Descazeaud , et al., “EAU Guidelines on the Treatment and Follow‐Up of Non‐Neurogenic Male Lower Urinary Tract Symptoms Including Benign Prostatic Obstruction,” European Urology 64, no. 1 (2013): 118–140. 23541338 10.1016/j.eururo.2013.03.004

[5] C. Bhanu , I. Petersen , M. Orlu , et al., “Drug‐Induced Orthostatic Hypotension: Cluster Analysis of Co‐Prescription Patterns in Older People in UK Primary Care,” Pharmacoepidemiology and Drug Safety 33, no. 1 (2024): e5730. 37974394 10.1002/pds.5730

[6] W. F. Gellad , J. L. Grenard , and Z. A. Marcum , “A Systematic Review of Barriers to Medication Adherence in the Elderly: Looking Beyond Cost and Regimen Complexity,” American Journal of Geriatric Pharmacotherapy 9, no. 1 (2011): 11–23. 21459305 10.1016/j.amjopharm.2011.02.004 PMC3084587

[7] D. Mosshammer , H. Haumann , K. Mörike , and S. Joos , “Polypharmacy—An Upward Trend With Unpredictable Effects,” Deutsches Ärzteblatt International 113, no. 38 (2016): 627–633. 27743469 10.3238/arztebl.2016.0627 PMC5078862

[8] M. Hennenberg , C. G. Stief , and C. Gratzke , “Prostatic Alpha1‐Adrenoceptors: New Concepts of Function, Regulation, and Intracellular Signaling,” Neurourology and Urodynamics 33, no. 7 (2014): 1074–1085. 24038148 10.1002/nau.22467

[9] M. Hennenberg , A. Acevedo , N. Wiemer , et al., “Non‐Adrenergic, Tamsulosin‐Insensitive Smooth Muscle Contraction Is Sufficient to Replace Alpha1‐Adrenergic Tension in the Human Prostate,” Prostate 77, no. 7 (2017): 697–707. 28116771 10.1002/pros.23293

[10] Y. Wang , T. Kunit , A. Ciotkowska , et al., “Inhibition of Prostate Smooth Muscle Contraction and Prostate Stromal Cell Growth by the Inhibitors of Rac, NSC23766 and EHT1864,” British Journal of Pharmacology 172, no. 11 (2015): 2905–2917. 25631101 10.1111/bph.13099 PMC4439884

[11] Q. Yu , C. Gratzke , Y. Wang , et al., “New Strategies for Inhibition of Non‐Adrenergic Prostate Smooth Muscle Contraction by Pharmacologic Intervention,” Prostate 79, no. 7 (2019): 746–756. 30811062 10.1002/pros.23780

[12] A. Herlemann , P. Keller , M. Schott , et al., “Inhibition of Smooth Muscle Contraction and ARF6 Activity by the Inhibitor for Cytohesin GEFs, SecinH3, in the Human Prostate,” American Journal of Physiology Renal Physiology 314, no. 1 (2018): F47–F57. 28855187 10.1152/ajprenal.00125.2017

[13] Q. Yu , C. Gratzke , Y. Wang , et al., “Inhibition of Human Prostate Smooth Muscle Contraction by the LIM Kinase Inhibitors, SR7826 and LIMKi3,” British Journal of Pharmacology 175, no. 11 (2018): 2077–2096. 29574791 10.1111/bph.14201 PMC5978953

[14] Q. Yu , C. Gratzke , Y. Wang , et al., “Inhibition of Prostatic Smooth Muscle Contraction by the Inhibitor of G Protein‐Coupled Receptor Kinase 2/3, CMPD101,” European Journal of Pharmacology 831 (2018): 9–19. 29698717 10.1016/j.ejphar.2018.04.022

[15] Y. Wang , C. Gratzke , A. Tamalunas , et al., “P21‐Activated Kinase Inhibitors FRAX486 and IPA3: Inhibition of Prostate Stromal Cell Growth and Effects on Smooth Muscle Contraction in the Human Prostate,” PLoS One 11, no. 4 (2016): e0153312. 27071060 10.1371/journal.pone.0153312 PMC4829229

[16] B. Li , X. Wang , R. Wang , et al., “Inhibition of Neurogenic and Thromboxane A2‐Induced Human Prostate Smooth Muscle Contraction by the Integrin α2β1 Inhibitor BTT‐3033 and the Integrin‐Linked Kinase Inhibitor Cpd22,” Prostate 80, no. 11 (2020): 831–849. 32449814 10.1002/pros.23998

[17] R. Huang , B. Li , A. Tamalunas , R. Waidelich , C. G. Stief , and M. Hennenberg , “Inhibition of Neurogenic Contractions in Renal Arteries and of Cholinergic Contractions in Coronary Arteries by the Presumed Inhibitor of ADP‐Ribosylation Factor 6, NAV2729,” Naunyn‐Schmiedeberg's Archives of Pharmacology 395, no. 4 (2022): 471–485. 35141760 10.1007/s00210-022-02218-2 PMC8873054

[18] S. Hu , G. Liu , O. Kale , et al., “Antagonism of Prostate α(1A)‐Adrenoceptors by Verapamil in Human Prostate Smooth Muscle Contraction,” Journal of Pharmacology and Experimental Therapeutics 392, no. 7 (2025): 103603. 40505412 10.1016/j.jpet.2025.103603 PMC12405930

[19] S. Hu , A. E. Müderrisoglu , A. Ciotkowska , et al., “Effects of Carvedilol on Human Prostate Tissue Contractility and Stromal Cell Growth Pointing to Potential Clinical Implications,” Pharmacological Reports 76, no. 4 (2024): 807–822. 38858312 10.1007/s43440-024-00605-5 PMC11294394

[20] S. P. H. Alexander , J. A. Cidlowski , E. Kelly , et al., “The Concise Guide to PHARMACOLOGY 2023/24: G Protein‐Coupled Receptors,” British Journal of Pharmacology 180, no. Suppl 2 (2023): S23–S144. 38123151 10.1111/bph.16177 PMC13324819

[21] C. Onesto , A. Shutes , V. Picard , F. Schweighoffer , and C. der , “Characterization of EHT 1864, a Novel Small Molecule Inhibitor of Rac Family Small GTPases,” Methods in Enzymology 439 (2008): 111–129. 18374160 10.1016/S0076-6879(07)00409-0

[22] A. Shutes , C. Onesto , V. Picard , B. Leblond , F. Schweighoffer , and C. J. der , “Specificity and Mechanism of Action of EHT 1864, a Novel Small Molecule Inhibitor of Rac Family Small GTPases,” Journal of Biological Chemistry 282, no. 49 (2007): 35666–35678. 17932039 10.1074/jbc.M703571200

[23] H. Akbar , J. Cancelas , D. A. Williams , J. Zheng , and Y. Zheng , “Rational Design and Applications of a Rac GTPase‐Specific Small Molecule Inhibitor,” Methods in Enzymology 406 (2006): 554–565. 16472687 10.1016/S0076-6879(06)06043-5

[24] Y. Gao , J. B. Dickerson , F. Guo , J. Zheng , and Y. Zheng , “Rational Design and Characterization of a Rac GTPase‐Specific Small Molecule Inhibitor,” Proceedings of the National Academy of Sciences of the United States of America 101, no. 20 (2004): 7618–7623. 15128949 10.1073/pnas.0307512101 PMC419655

[25] R. Wang , Q. Yu , X. Wang , et al., “Rac1 Silencing, NSC23766 and EHT1864 Reduce Growth and Actin Organization of Bladder Smooth Muscle Cells,” Life Sciences 261 (2020): 118468. 32961232 10.1016/j.lfs.2020.118468

[26] M. Levay , K. A. Krobert , K. Wittig , et al., “NSC23766, a Widely Used Inhibitor of Rac1 Activation, Additionally Acts as a Competitive Antagonist at Muscarinic Acetylcholine Receptors,” Journal of Pharmacology and Experimental Therapeutics 347, no. 1 (2013): 69–79. 23887096 10.1124/jpet.113.207266

[27] B. Li , Q. Yu , R. Wang , et al., “Inhibition of Female and Male Human Detrusor Smooth Muscle Contraction by the Rac Inhibitors EHT1864 and NSC23766,” Frontiers in Pharmacology 11 (2020): 409. 32317972 10.3389/fphar.2020.00409 PMC7154109

[28] M. Hafner , A. Schmitz , I. Grüne , et al., “Inhibition of Cytohesins by SecinH3 Leads to Hepatic Insulin Resistance,” Nature 444, no. 7121 (2006): 941–944. 17167487 10.1038/nature05415

[29] A. M. Hayallah , “Design and Synthesis of New (SecinH3) Derivatives as Potential Cytohesin Inhibitors,” Indian Journal of Pharmaceutical Sciences 76, no. 5 (2014): 387–400. 25425752 PMC4243255

[30] Y. Yin , K. Zheng , N. Eid , et al., “Bis‐Aryl Urea Derivatives as Potent and Selective LIM Kinase (Limk) Inhibitors,” Journal of Medicinal Chemistry 58, no. 4 (2015): 1846–1861. 25621531 10.1021/jm501680m PMC4349585

[31] P. Ross‐Macdonald , H. de Silva , Q. Guo , et al., “Identification of a Nonkinase Target Mediating Cytotoxicity of Novel Kinase Inhibitors,” Molecular Cancer Therapeutics 7, no. 11 (2008): 3490–3498. 19001433 10.1158/1535-7163.MCT-08-0826

[32] S. Ikeda , M. Keneko , and S. Fujiwara , “Cardiotonic Agent Comprising GRK Inhibitor,” US Patent, (2007).

[33] D. M. Thal , R. Y. Yeow , C. Schoenau , J. Huber , and J. J. G. Tesmer , “Molecular Mechanism of Selectivity Among G Protein‐Coupled Receptor Kinase 2 Inhibitors,” Molecular Pharmacology 80, no. 2 (2011): 294–303. 21596927 10.1124/mol.111.071522 PMC3141885

[34] J. D. Lowe , H. S. Sanderson , A. E. Cooke , et al., “Role of G Protein‐Coupled Receptor Kinases 2 and 3 in Mu‐Opioid Receptor Desensitization and Internalization,” Molecular Pharmacology 88, no. 2 (2015): 347–356. 26013542 10.1124/mol.115.098293 PMC4518089

[35] B. M. Dolan , S. G. Duron , D. A. Campbell , et al., “Rescue of Fragile X Syndrome Phenotypes in Fmr1 KO Mice by the Small‐Molecule PAK Inhibitor FRAX486,” Proceedings of the National Academy of Sciences of the United States of America 110, no. 14 (2013): 5671–5676. 23509247 10.1073/pnas.1219383110 PMC3619302

[36] S. L. Lee , E. C. Hsu , C. C. Chou , et al., “Identification and Characterization of a Novel Integrin‐Linked Kinase Inhibitor,” Journal of Medicinal Chemistry 54, no. 18 (2011): 6364–6374. 21823616 10.1021/jm2007744 PMC3182772

[37] M. C. Michel , T. J. Murphy , and H. J. Motulsky , “New Author Guidelines for Displaying Data and Reporting Data Analysis and Statistical Methods in Experimental Biology,” Molecular Pharmacology 97, no. 1 (2020): 49–60. 31882404 10.1124/mol.119.118927

[38] S. D. Harding , J. L. Sharman , E. Faccenda , et al., “The IUPHAR/BPS Guide to PHARMACOLOGY in 2018: Updates and Expansion to Encompass the New Guide to IMMUNOPHARMACOLOGY,” Nucleic Acids Research 46, no. D1 (2018): D1091–d1106. 29149325 10.1093/nar/gkx1121 PMC5753190

[39] S. P. H. Alexander , J. A. Cidlowski , E. Kelly , et al., “The Concise Guide to PHARMACOLOGY 2023/24: Enzymes,” British Journal of Pharmacology 180, no. Suppl 2 (2023): S289–s373. 38123154 10.1111/bph.16181

[40] S. P. Alexander , E. Kelly , A. A. Mathie , et al., “The Concise Guide to PHARMACOLOGY 2023/24: Introduction and Other Protein Targets,” British Journal of Pharmacology 180, no. Suppl 2 (2023): S1–s22. 38123153 10.1111/bph.16176 PMC13480408

[41] B. Li , R. Huang , R. Wang , Y. Liu , C. G. Stief , and M. Hennenberg , “Picotamide Inhibits a Wide Spectrum of Agonist‐Induced Smooth Muscle Contractions in Porcine Renal Interlobar and Coronary Arteries,” Pharmacology Research & Perspectives 9, no. 3 (2021): e00771. 33929093 10.1002/prp2.771 PMC8085950

[42] L. Désiré , J. Bourdin , N. Loiseau , et al., “RAC1 Inhibition Targets Amyloid Precursor Protein Processing by Gamma‐Secretase and Decreases Abeta Production In Vitro and In Vivo,” Journal of Biological Chemistry 280, no. 45 (2005): 37516–37525. 16150730 10.1074/jbc.M507913200

[43] Y. Enomoto , T. Onuma , T. Hori , et al., “Synergy by Ristocetin and CXCL12 in Human Platelet Activation: Divergent Regulation by Rho/Rho‐Kinase and Rac,” International Journal of Molecular Sciences 24, no. 11 (2023): 9716. 37298667 10.3390/ijms24119716 PMC10253402

[44] S. Dütting , J. Heidenreich , D. Cherpokova , et al., “Critical Off‐Target Effects of the Widely Used Rac1 Inhibitors NSC23766 and EHT1864 in Mouse Platelets,” Journal of Thrombosis and Haemostasis 13, no. 5 (2015): 827–838. 25628054 10.1111/jth.12861

[45] A. Egyed , D. J. Kiss , and G. M. Keseru , “The Impact of the Secondary Binding Pocket on the Pharmacology of Class A GPCRs,” Frontiers in Pharmacology 13 (2022): 847788. 35355719 10.3389/fphar.2022.847788 PMC8959758

[46] H. Lin , M. F. Sassano , B. L. Roth , and B. K. Shoichet , “A Pharmacological Organization of G Protein‐Coupled Receptors,” Nature Methods 10, no. 2 (2013): 140–146. 23291723 10.1038/nmeth.2324 PMC3560304

[47] R. Huang , Y. Liu , A. Ciotkowska , et al., “Concentration‐Dependent alpha1‐Adrenoceptor Antagonism and Inhibition of Neurogenic Smooth Muscle Contraction by Mirabegron in the Human Prostate,” Frontiers in Pharmacology 12 (2021): 666047. 34248624 10.3389/fphar.2021.666047 PMC8264149

[48] M. C. Michel , E. Arioglu‐Inan , and M. Hennenberg , “beta(3)‐Adrenoceptor Agonist Effects on the Urinary Bladder Beyond Detrusor Relaxation,” Neurourology and Urodynamics 44 (2025): 1498–1502. 40159930 10.1002/nau.70043 PMC12319471

[49] B. Li , R. Wang , Y. Wang , C. G. Stief , and M. Hennenberg , “Regulation of Smooth Muscle Contraction by Monomeric Non‐RhoA GTPases,” British Journal of Pharmacology 177, no. 17 (2020): 3865–3877. 32579705 10.1111/bph.15172 PMC7429483

[50] S. Qian , S. Hu , W. Zhu , A. Tamalunas , C. G. Stief , and M. Hennenberg , “Silencing of Rac1 and Arf6 Reduces Time‐Dependent and Carbachol‐Induced Contractions, Proliferation, Survival and Growth in Human Bladder Smooth Muscle Cells,” World Journal of Urology 43 (2025): 279. 40332567 10.1007/s00345-025-05652-y PMC12058880

[51] A. P. Harvey , A. C. Montezano , K. Y. Hood , et al., “Vascular Dysfunction and Fibrosis in Stroke‐Prone Spontaneously Hypertensive Rats: The Aldosterone‐Mineralocorticoid Receptor‐Nox1 Axis,” Life Sciences 179 (2017): 110–119. 28478264 10.1016/j.lfs.2017.05.002 PMC5446265

[52] P. Kücük , L. Abbey , J. Schmitt , C. Henninger , and G. Fritz , “Cardiomyocytes, Cardiac Endothelial Cells and Fibroblasts Contribute to Anthracycline‐Induced Cardiac Injury Through RAS‐Homologous Small GTPases RAC1 and CDC42,” Pharmacological Research 203 (2024): 107165. 38561112 10.1016/j.phrs.2024.107165

[53] C. Yao , X. Fang , Q. Ru , et al., “Tiam1‐Mediated Maladaptive Plasticity Underlying Morphine Tolerance and Hyperalgesia,” Brain 147, no. 7 (2024): 2507–2521. 38577773 10.1093/brain/awae106 PMC11224607

[54] Y. Guo , J. Xiong , J. Wang , J. Wen , and F. Zhi , “Inhibition of Rac Family Protein Impairs Colitis and Colitis‐Associated Cancer in Mice,” American Journal of Cancer Research 8, no. 1 (2018): 70–80. 29416921 PMC5794722

[55] S. Kinsella , C. A. Evandy , K. Cooper , et al., “Attenuation of Apoptotic Cell Detection Triggers Thymic Regeneration After Damage,” Cell Reports 37, no. 1 (2021): 109789. 34610317 10.1016/j.celrep.2021.109789 PMC8627669

[56] S. M. Marques , M. R. Melo , D. B. Zoccal , et al., “Acute Inhibition of Nicotinamide Adenine Dinucleotide Phosphate Oxidase in the Commissural Nucleus of the Solitary Tract Reduces Arterial Pressure and Renal Sympathetic Nerve Activity in Renovascular Hypertension,” Journal of Hypertension 41, no. 10 (2023): 1634–1644. 37466439 10.1097/HJH.0000000000003516

[57] H. H. Hassanain , D. Gregg , M. L. Marcelo , et al., “Hypertension Caused by Transgenic Overexpression of Rac1,” Antioxidants & Redox Signaling 9, no. 1 (2007): 91–100. 17115888 10.1089/ars.2007.9.91

[58] G. André , J. E. Sandoval , K. Retailleau , et al., “Smooth Muscle Specific Rac1 Deficiency Induces Hypertension by Preventing p116RIP3‐Dependent RhoA Inhibition,” Journal of the American Heart Association 3, no. 3 (2014): e000852. 24938713 10.1161/JAHA.114.000852 PMC4309079

[59] Q. Yu , C. Gratzke , R. Wang , et al., “A NAV2729‐Sensitive Mechanism Promotes Adrenergic Smooth Muscle Contraction and Growth of Stromal Cells in the Human Prostate,” Journal of Biological Chemistry 294, no. 32 (2019): 12231–12249. 31243101 10.1074/jbc.RA119.007958 PMC6690707

[60] R. Wang , S. Schneider , O. T. Keppler , et al., “ADP Ribosylation Factor 6 Promotes Contraction and Proliferation, Suppresses Apoptosis and Is Specifically Inhibited by NAV2729 in Prostate Stromal Cells,” Molecular Pharmacology 100, no. 4 (2021): 356–371. 34349027 10.1124/molpharm.121.000304

[61] B. R. Erdogan and M. C. Michel , “Does Coupling to ADP Ribosylation Factor 6 Explain Differences Between Muscarinic and Other Receptors in Interaction With Beta‐Adrenoceptor‐Mediated Smooth Muscle Relaxation?,” Naunyn‐Schmiedeberg's Archives of Pharmacology 395, no. 4 (2022): 381–386. 35175382 10.1007/s00210-022-02221-7 PMC8873149

[62] Q. Yu , C. Wu , Y. Chen , et al., “Inhibition of LIM Kinase Reduces Contraction and Proliferation in Bladder Smooth Muscle,” Acta Pharmaceutica Sinica B 11, no. 7 (2021): 1914–1930. 34386328 10.1016/j.apsb.2021.01.005 PMC8343115

[63] Y. Shen , D. A. Goncharov , T. Avolio , et al., “Differential Effects of Integrin‐Linked Kinase Inhibitor Cpd22 on Severe Pulmonary Hypertension in Male and Female Rats,” Pulmonary Circulation 10, no. 1 (2020): 2045894019898593. 32110386 10.1177/2045894019898593 PMC7016388

