# Supplementary material for: Vasoactivity of Rac GTPase, Cytohesin and Kinase Inhibitors in Renal Interlobar and Coronary Arteries Reveals Shared and Distinct Patterns of Inhibitory Effects in Vascular and Prostate Smooth Muscle Contraction
Source: Pharmacol Res Perspect. 2025 Nov 18;13(6):e70190. doi: 10.1002/prp2.70190 (PMC12626100; doi:10.1002/prp2.70190)
Supplement: Supplementary file 1 — Table S1: P values from Shapiro‐Wilk W tests, for assessment of residuals of concentration response and frequency response curves for normality. P values ≥ 0.05 suggest normal distribution. Compare main text for limitations and details. [file PRP2-13-e70190-s001.pdf]

Renal arteries

p values from *Shapiro-Wilk W*
note: p values ≥0.05 suggest normal distribution (see main text for limitations)

			p for normality of residuals
EHT1864	NA	control	
		100 µM	
	PE	control	
		100 µM	< 0.0001
	MTX	control	0.0011
		100 µM	0.0001
	ET-1	control	0.001
		100 µM	< 0.0001
	U46619	control	0.307
		100 µM	0.3695
	EFS	control	0.0661
		100 µM	0.0028
NSC23766	NA	control	< 0.0001
		100 µM	0.001
	PE	control	0.8943
		100 µM	0.022
	MTX	control	0.9908
		100 µM	< 0.0001
	ET-1	control	< 0.0001
		100 µM	0.5482
	U46619	control	0.032
		100 µM	0.0003
	EFS	control	0.0002
		100 µM	0.0219
SecinH3	NA	control	0.0701
		30 µM	< 0.0001
	PE	control	< 0.0001
		30 µM	0.1265
	EFS	control	0.053
		30 µM	< 0.0001
SR7826	NA	control	< 0.0001
		1 µM	0.026
	PE	control	< 0.0001
		1 µM	0.0147
	MTX	control	0.0606
		1 µM	< 0.0001
	ET-1	control	0.8968
		1 µM	0.0195
	U46619	control	0.006
		1 µM	0.7011
	EFS	control	0.6673
		1 µM	n.a. (interrupted)
LIMKi3	NA	control	n.a. (interrupted)
		30 µM	0.0388
	PE	control	0.486
		30 µM	0.0051
	EFS	control	0.0207
		30 µM	0.0708
CMPD101	NA	control	0.9054
		50 µM	0.1212
	PE	control	0.2829
		50 µM	0.0017
	MTX	control	0.0049
		50 µM	0.0167
	ET-1	control	0.0015
		50 µM	0.0065

EHT1864	PE	control	< 0.0001
		10 µM	< 0.0001
		control	0.001
		1 µM	0.0003
	EFS	control	< 0.0001
		10 µM	< 0.0001
		control	0.0003
		1 µM	0.0549
NSC23766	PE	control	0.0125
		10 µM	0.0253
		control	0.0035
		1 µM	0.0003
	EFS	control	0.162
		10 µM	0.0414
		control	0.0004
		1 µM	0.5558

	U46619	control	< 0.0001
		50 µM	0.0002
	EFS	control	0.0374
		50 µM	0.0051
	NA	control	n.a. (not converged)
		30 µM	0.0075
FAX486	PE	control	0.023
		30 µM	0.0015
	EFS	control	0.0255
		30 µM	n.a. (interrupted)
	NA	control	< 0.0001
		3 µM	0.1814
Cpd22	PE	control	0.0025
		3 µM	0.0318
	EFS	control	< 0.0001
		3 µM	0.0144
			< 0.0001
			0.002
			0.1481

Coronary Arteries

p values from *Shapiro-Wilk W*
note: p values ≥0.05 suggest normal distribution (see main text for limitations)

			p for normality of residuals
		control	0.0076
	carbachol	100 µM	< 0.0001
		control	0.0964
		10 µM	n.a. (not converged)
EHT1864		control	0.1199
	methacholine	100 µM	0.2281
		control	< 0.0001
		10 µM	0.0593
		control	0.7687
	carbachol	100 µM	0.001
		control	< 0.0001
		10 µM	0.0004
NSC23766		control	0.1311
	methacholine	100 µM	0.1392
		control	< 0.0001
		10 µM	0.0122
		control	0.0009
	carbachol	30 µM	0.0678
		control	0.2317
		10 µM	0.0129
SecinH3		control	< 0.0001
	methacholine	30 µM	n.a. (interrupted)
		control	< 0.0001
		10 µM	0.0235
